# National Hypertension Guidelines: A Review of the India Hypertension Control Initiative (IHCI) and Future Prospects

**DOI:** 10.7759/cureus.27997

**Published:** 2022-08-14

**Authors:** Swedaj Thakre, Ashish Anjankar, Arihant Singh, Tanishq Kumar

**Affiliations:** 1 Medicine, Jawaharlal Nehru Medical College, Datta Meghe Institute of Medical Sciences, Wardha, IND; 2 Biochemistry, Jawaharlal Nehru Medical College, Datta Meghe Institute of Medical Sciences, Wardha, IND

**Keywords:** hypertension management and treatment, simple app, indian council of medical research, india, national family health survey, who-india, national guidelines, hypertension

## Abstract

Hypertension is a silent killer; however, the treatment of hypertension is simple, effective, readily available, and needs to be continued lifelong. It is a significant health problem that is included under the umbrella of non-communicable disease conditions and has a strong alliance with cardiovascular morbidity and mortality. The India Hypertension Control Initiative (IHCI) is an extensive program in India that involves the Indian Council of Medical Research, the Ministry of Health and Family Welfare (Government of India), the state governments of India, and World Health Organization Country Office for India (WHO-India). The IHCI is a multi-partner initiative carried forward systemically across various states. The states are categorized into Grade I and Grade II. There is the involvement of specialized teams of cardiovascular experts and health officials to insure precise execution and seamless healthcare service.

The implementation of the free and easy-to-use mobile application and software, Simple (Resolve to Save Lives, New York City, United States), in the analysis and storage of data, is a novel step taken to insure safe record keeping and follow-ups. Emphasis is on the adoption of demography-specific interventional methods and drugs, and proper acquisition and storage of these drugs is the key step. Treatment modalities involve the adoption of medicines and lifestyle modifications as a combined recipe. Advancements have been made in the area of drug development like gene therapies but they seem to show low success rates at the given moment. Adoption of lifestyle modifications along with medications is the gold standard treatment option. This review article aims to shed light on the current status of IHCI, its milestones, and the future of the initiative in India.

## Introduction and background

Hypertension is the biggest cause of mortality in the world and global mortality; this global caseload is expected to increase five-fold by the year 2030 [1-3]. It is a disease characterized by excessive pressure exerted by the ejecting blood on the vessel walls. Hypertension causes multiple cardiac complications such as stroke, ischemia, angina, and tamponade. Various studies suggest that hypertension is increasing and that there is a need for awareness and control [4]. The fourth National Family Health Survey conducted in 2015-16 in India concluded that hypertension was more prevalent among men (27.4%) than women (20.0%); roughly estimated to be 112 million men and 95 million women in the country. Hypertension was the sole responsible factor that led to almost 1.64 million deaths in India according to the Global Burden of Diseases study of 2016 [5,6].

Risk factors for hypertension are numerous. They are characterized into non-modifiable and modifiable types. The non-modifiable factors mainly consist of age, sex, gender, race, ethnicity, and genetic predisposition. Modifiable risk factors, on the other hand, are the ones that contribute the most to increasing the case burden and involve blood lipid profiling, weight/obesity, lifestyle, and eating habits; these are the ones that can easily be avoided if proper care and precautions are in place [7]. Improper dietary habits rich in saturated fats and high sodium content increases the risk of hypertension. This along with lack of mobility and exercise, and improper hygiene, just adds more fuel to the fire of this non-communicable disease. Cigarette smoking is also on the list of modifiable factors [8]. The cardiovascular system (CVS) is the most commonly affected system and can progress to cause complications like atherosclerotic plaques, coronary disease, and ischemic heart diseases. Other systems soon get involved as the CVS gets affected and starts with pulmonary complications and leads to the organs with hematological significance [9,10]. 

A study conducted by Yip and his colleagues in 2013 tried to estimate the amount of Incidence, control, and awareness of hypertension among Indians living in urban Singapore and rural India. The result was analyzed from the data of population-based studies conducted in their respective regions. This study reflected that the incidence, control, and awareness of hypertension were higher in Indians living in urban Singapore as opposed to rural India. Factors like socioeconomic background and other metabolic factors were the reasons for this difference between these two populations. This study reflected that modifiable risk factors were the primary causative agents for hypertension [11,12]. 

The India Hypertension Control Initiative (IHCI) is a program led by the World Health Organization Country Office for India (WHO-India) as a high-impact and low-cost solution for the control and treatment of hypertension and related conditions. The main goal of IHCI is to fast-track access to treatment services. It was initiated in 2017 in a phase-wise manner and has set a target of a 20-25% relative reduction in the prevalence of hypertension by 2025 [13,14]. 

IHCI is a multi-partner initiative that involves the Government of India's Ministry of Health & Family Welfare, the Indian Council of Medical Research (ICMR), WHO-India, and Resolve to Save Lives (technical partnership).

This review aims to shed light on IHCI, which is the latest program that is setting significant benchmarks in the rural community of India. The assessment is done by comparing the data from the NFHS from its 4th iteration all the way to monthly and yearly progress reports from this WHO-led initiative. The data is directly taken from the national reports released by the agencies backing the surveys and can be subjected to scrutiny for its limitations. The use of the Simple App in this program is a novel step in the management of patient data. This review also aims to raise awareness regarding various control measures for the prevention and management of hypertension among healthcare professionals and the general public.

## Review

The NFHS is a gigantic and legion survey conducted amongst a significant sample of families throughout our country. It is responsible for the collection of essential data, which acts as an indicator for policymakers to induce reforms and policies for the greater good. Five rounds of this humongous survey have taken place from 1992-93. The survey sheds light on the information on fertility, child and infant mortality, family planning, maternal and child health, nutrition, reproductive health, anemia, and the quality and the delivery of the health and family planning services. The specific goals of the NFHS include gathering valuable information on health and family welfare that will be analyzed by the health ministry and other branches of the government for creating steps of intervention and to shed light on newly trending and alarming health and family issues. The International Institute for Population Sciences (IIPS), Mumbai, India, is appointed as the leading organization by the Ministry of Health for providing technical support for the survey. IIPS then collaborated with numerous local organizations for insurance of proper implementation of the survey. Other technical partners involved include ICF Incorporated, United States. Funding for different rounds was provided by United Nations Children's Fund (UNICEF), United States Agency for International Development (USAID), United Nations Population Fund (UNFPA), Bill & Melinda Gates Foundation, and MoHFW [15].

These family health surveys and the data gathered from various rounds will provide significant insight into the incidence and pervasiveness of non-communicable diseases like hypertension in the general population. The data is significant for understanding the epidemiology of the disease condition so that strategic plans can be laid out for implementing prevention measures.

Data from NFHS-4

NFHS-4 was the fourth round of the national family health survey in India that facilitated information regarding the population of the country, their health, and nutrition in 2015-16. The primary objective of the survey was to gather essential information on health and family welfare, as well as information on emerging concerns in these areas. The clinical and anthropometric tools used in the study were designed to provide vital approximates of the incidence of problems like hypertension through a series of tests and measurements.

**Figure 1:**
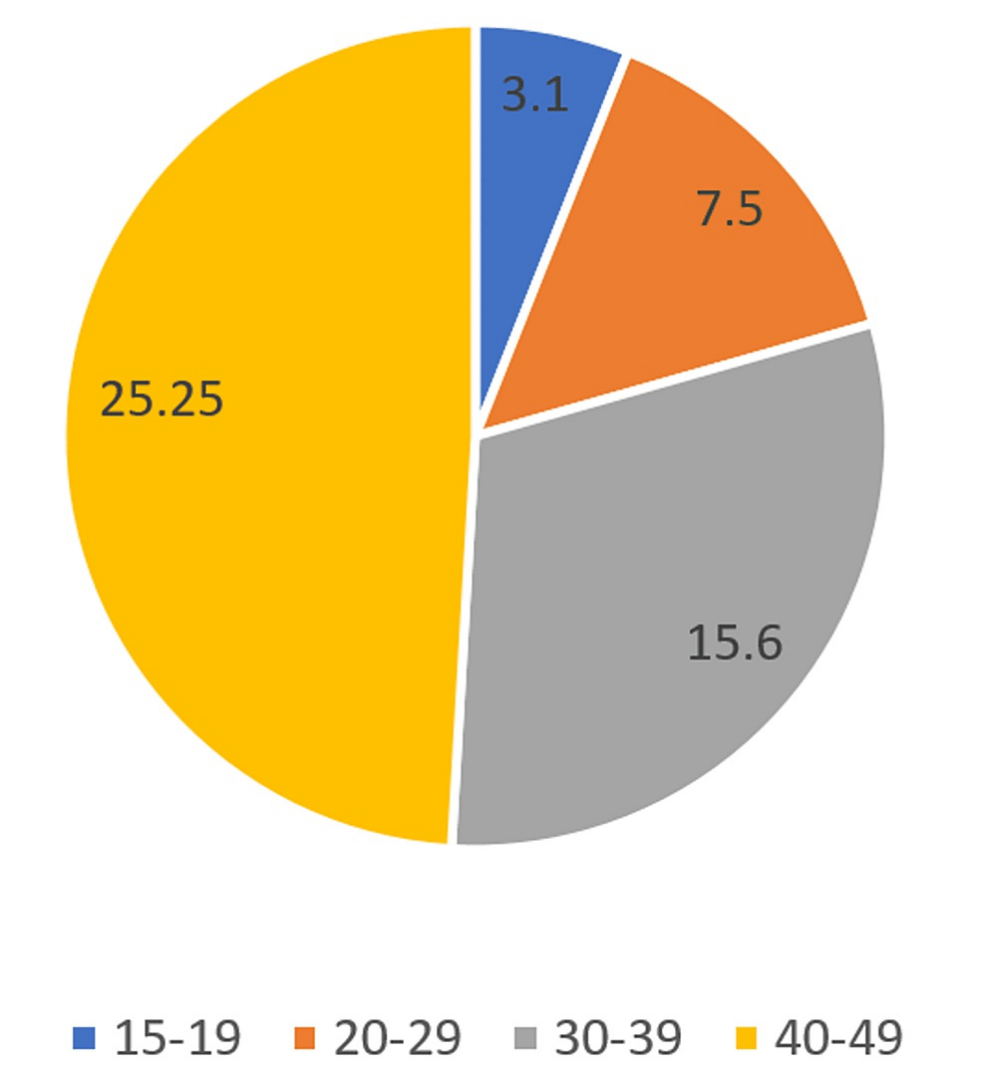
Pie-chart describing average incidence of hypertension as per age from NFHS-4 NFHS-4: National Family Health Survey 4

Sample: Male and female individuals aged 15-49. The key findings in the study indicated that 10% of the female population had hypertension, including 8% with stage-1 and 1% each with stage-2 and stage-3 hypertension; 61% of women have normal blood pressure and almost one-third of women are pre-hypertensive. Only 1% of women affected are taking hypertension medications and have their blood pressure in check. The incidence of hypertension among the male population is higher; 15% have hypertension, which includes 10% of people with stage 1 hypertension, 2% with stage 2 hypertension, and 1% with stage-3 hypertension. Of the male population, 42% have normal blood pressure and the same percentage is pre-hypertensive. Only 1% of hypertensive males are taking medication and have their blood pressure in check. It was also captured that the incidence of hypertension increased exponentially with an increase in age for both male and female populations. The incidence is higher among the Sikh population, followed by the Jain and Buddhist/Neo-Buddhist populations than the rest of the religious groups. There is the occurrence of hypertension among people with BMI with hypertension ubiquitous among 29% of obese females and 37% of obese males. The north-eastern population showed a higher caseload as compared to the national average [16]. Figure 1 describes the average distribution as per age group in both males and females according to NFHS-4.

Data from NFHS-5

NFHS-5 was the fifth edition of the national survey, conducted by the MoHFW in 2019-2021; this study was a continuation of the NFHS-4 from 2015-2016. The age range for blood pressure estimation was stretched to accommodate more valuable input.

**Figure 2:**
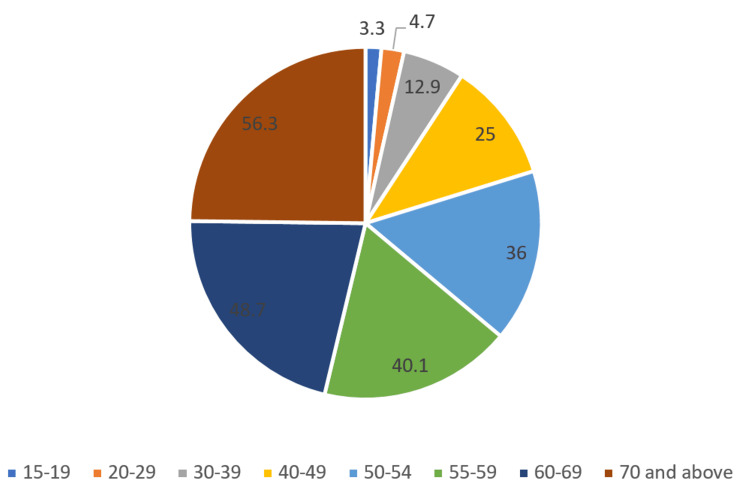
Pie-chart describing average incidence of hypertension as per age groups from NFHS-5 NFHS-5: National Family Health Survey 5

Sample: Male and female population of ages 15 and above. The findings of the study indicated that 21% of women aged 15 and above had hypertension, which included 11% with mildly elevated blood pressure, 4% with moderately elevated blood pressure, and 2% with severely elevated blood pressure. Of the female population, 44% have normal blood pressure, with almost two-fifths, i.e., 39% of the female population being pre-hypertensive. Only 1% of the affected population were taking hypertension medication. The incidence of hypertension among the male population is higher compared to the female population; 24% of men above 15 years of age have hypertension, including 16% with mildly elevated blood pressure, 4% with moderately elevated blood pressure, and 2% with severely elevated blood pressure. While 29% of the men are unaffected, 48% are pre-hypertensive. Only 1% of men are currently taking medication for hypertension. The trend of increment of caseload with the growing age is very much prevalent and is applicable for all categories of disease states for both male and female populations. The incidence of cases is higher among Sikhs, followed by Jains and Christians than the rest of the religions. The connection of BMI with hypertension is very much prevalent in this study for both genders; hypertension was prevalent among 40% of overweight men and 28% of overweight women. The state of Sikkim had the highest number of female and male cases [17]. Figure 2 describes the average distribution as per age group according to NFHS-5.

Working of the IHCI

The IHCI is a joint initiative that was implemented in November 2017. The mission statement of the program is to reduce the issue of hypertension by 25% by 2025. The initiative is backed by the Government of India's MoHFW, ICMR, and WHO-India with Resolve to Save Lives as an international technical partner.

The main goal is to build up the treatment infrastructure and provide smooth follow-ups for the patients of the National Program for Prevention and Control of Cancer, Diabetes, Cardiovascular Diseases, and Stroke (NPCDCS). The IHCI covered 26 districts across the states of Punjab, Kerala, Madhya Pradesh, Telangana, and Maharashtra in Phase-I (2017). The initiative expanded its reach in the next two years to include the districts of Rajasthan, Gujarat, Utter Pradesh, Jharkhand, West Bengal, Karnataka, Goa, Andhra Pradesh, Tamil Nadu, Bihar, Sikkim, and Nagaland; These states were termed as the Phase-II states. The key strategies used by the IHCI included setting up of protocols, provision and maintenance of medical supply, formation of teams and division of tasks between them, implementation of patient-centric care along with introduction and management of information systems for the storage of the patient data for follow-ups and progress keeping [18,19].

The key intervention strategies involve the use of state-specific standard drugs and dose-specific algorithms for hypertension management, an uninterrupted supply of the prescribed drugs, the presence of well-trained staff at all levels, high-quality services using patient-centric approaches, team-based care for hypertension management along with proper monitoring systems, inculcation of a dedicated workforce for supportive supervision and monitoring that provides feedback to the stakeholders, decentralization of patients by implementing health and wellness retreats for follow-ups, patient education, and prescription refills [20].

The Simple app

Simple is a fast and free mobile application that is available online around the world and was developed with the IHCI for doctors and officials to manage patients with hypertension and diabetes. Using this android app, doctors are able to save and manage essential patient data, including blood pressure and blood glucose levels, as well as recordings and scheduling of medicines and follow-up visitations. It also includes an effortless web-based dashboard server that provides system managers with appropriate feedback to improve the management of services. The patient can use this app to chart their progress and get daily scheduled notifications to remind them about to get their medicines [21]. 

When the coronavirus disease 2019 (COVID-19) pandemic forced everyone to stay inside their homes in 2020, lakhs of people with non-communicable diseases like diabetes and hypertension were unable to access essential treatment. It was difficult even after the restrictions were lifted in a phase-wise manner due to social distancing. The fear of contracting the virus kept people away from Primary Health Centers (PHCs) and hospitals. The solution was then found from an advanced interventional initiative from IHCI. Under this program, 13 state authorities are assisted by a team of IHCI health officials and senior treatment supervisors from WHO-India to adopt demography-specific treatment options, smoothening the forecasting and acquisition of hypertension medication and implementation of cohort surveillance through effective information storage systems, including apps like Simple, a mobile application for the management of hypertension that was modified to include telemedicine features for the delivery of medicines at the community level [22]. 

Annual progress report of IHCI for 2021

The initiative was implemented in 101 districts across 19 states and had registered more than 200,000 patients across more than 12,000 health facilities, although the registration was slowed down due to the COVID-19 pandemic. Nearly one-fourth, i.e., almost 23% had uncontrolled blood pressure in spite of follow-ups at the facilities, and approximately 27% did not have a documented visit during the first quarter of 2021. Overall, the blood pressure control was at the highest at 55% at wellness centers and second highest at 48% at the PHCs. Community-level hypertension control also witnessed improvement in 21 project districts in 2021 instead of 2020. Among an estimated 4.6 million hypertensive cases across the initial phase-I 26 districts, blood pressure control incremented by more than four times. Overall, the number of patients with increased blood pressure control continued to increase over three years. There was a significant decline observed in Kerala, which was due to the poor documentation during the COVID-19 pandemic and the inability of the logistical services to supply adequate medicines.

Logistical infrastructure at the levels of districts experienced a significant boost owing to systematic planning, efficient acquisition of the drugs, and proper field-level surveillance. States like Madhya Pradesh, Punjab, and Telangana had drug stocks for almost six months. Maharashtra and Kerala had low drug stocks due to the pandemic wreaking havoc in full swing. Phase-II states suffered from the slow acquisition of the needed drugs and delayed application.

Decentralization of patients was a monumental adoption for creating a sustainable health delivery system during the COVID-19 pandemic. Certain refinements in the area of staffing, screening, logistics of the drugs, screening, and dependable database for the cases were needed to enhance the overall impact of the initiative [23].

Progress report of IHCI for January-May, 2022

"Mitanins" played a pivotal role in the enhancement of blood pressure control in the state of Chhattisgarh. The initiative was implemented in the month of March 2020. The community health workers called Accredited Social Health Activists (ASHA), are colloquially termed Mitanins in Chhattisgarh. They played a critical role in the tracking of the hypertensive patients and ensured regular follow-ups for the registered patients with screening and refilling of medications. IHCI made a rapid stride in the state of Rajasthan. The initiative paved the way for improved outcomes for hypertensive patients in the district of Charu and Bikaner. There are almost 40,000 patients enrolled under the IHCI program in the state, and the data provide indications that more than 50% of the patients have their blood pressure under control in only a short period of 14 months [24].

Regardless of the advancements made in recent years for screening, treatment, and prevention of elevated blood pressure, hypertension is still one of the toughest non-communicable diseases to look out for [25]. Nearly one billion people are affected worldwide due to this condition which causes an increase in the incidence of morbidity and mortality. It cannot be eradicated as there is no antidote or vaccine to prevent its pathophysiology, but its occurrence can be governed by reducing the exposure to risk factors [26]. Several studies and clinical trials suggest that lifestyle modifications and hypertension medications should be used in combination to fight this disease. Lifestyle modifications must be the dominant factor in this strategy with required drugs [27,28]. Gene therapy might also help in curing this condition, but it is still a hypothesis as much work is needed in the area. Numerous problems were encountered in the application of gene therapy, especially a high occurrence of adverse effects which made it a non-viable option. The study in animals showed a remarkable result as opposed to the difficulties in human applications, so there is hope for gene therapy [29]. Drug optimization along with lifestyle modifications is the way to go for now [30-35]. 

## Conclusions

There are steps in place for the advancement of IHCI project under the NPCDCS. IHCI is an ingenious approach for the management of cases of hypertension with the collaboration of community and technology and has proved its feasibility. The results validate the project as a practical option in PHC settings across the country. The IHCI is also provisioning free healthcare for all the hypertensive patients and other non-communicable diseases, which promises delivery of the services for the people in need.

The implementation of the Simple App as a data management tool is a step in the right direction. If this tool holds its value in the long run then similar initiatives can be adopted in the country for making the process of data collection more efficient. With authentic planning and execution, IHCI is a pioneering initiative in the control of hypertension in India.
